# Time Course of Reach Adaptation and Proprioceptive Recalibration during Visuomotor Learning

**DOI:** 10.1371/journal.pone.0163695

**Published:** 2016-10-12

**Authors:** Jennifer E. Ruttle, Erin K. Cressman, Bernard Marius ’t Hart, Denise Y. P. Henriques

**Affiliations:** 1 Centre for Vision Research, York University, Toronto, Canada; 2 Department of Psychology, York University, Toronto, Canada; 3 School of Kinesiology and Health Science, York University, Toronto, Canada; 4 School of Human Kinetics, University of Ottawa, Ottawa, Canada; Centre de neuroscience cognitive, FRANCE

## Abstract

Training to reach with rotated visual feedback results in adaptation of hand movements, which persist when the perturbation is removed (reach aftereffects). Training also leads to changes in felt hand position, which we refer to as proprioceptive recalibration. The rate at which motor and proprioceptive changes develop throughout training is unknown. Here, we aim to determine the timescale of these changes in order to gain insight into the processes that may be involved in motor learning. Following six rotated reach training trials (30° rotation), at three radially located targets, we measured reach aftereffects and perceived hand position (proprioceptive guided reaches). Participants trained with opposing rotations one week apart to determine if the original training led to any retention or interference. Results suggest that both motor and proprioceptive recalibration occurred in as few as six rotated-cursor training trials (7.57° & 3.88° respectively), with no retention or interference present one week after training. Despite the rapid speed of both motor and sensory changes, these shifts do not saturate to the same degree. Thus, different processes may drive these changes and they may not constitute a single implicit process.

## Introduction

Healthy participants adapt their reaching behavior when movement dynamics or visual feedback are altered, such as in velocity dependent force-fields [1, 2] or in visuomotor adaptation paradigms [3, 4, 5, 6, 7]. These changes in motor behavior persist even after the perturbation is removed, called reach aftereffects. In addition to reach aftereffects, our lab has repeatedly demonstrated that sensory changes accompany training with rotated visual feedback: the felt hand position shifts toward the visual feedback [8, 9, 10, 11]. Similar recalibration of felt hand motion also occurs after force-field training [12, 13]. Limited information is available on how quickly reach aftereffects and proprioception arise and how persistent they are across longer time periods. Here we set out to characterize both reach aftereffects and sensory changes in visuomotor adaptation. Using temporally dense sampling, we will be able to establish how quickly these two processes first arise, as well as quantify retention and interference after a week of no training.

While reach aftereffects and proprioceptive recalibration have been investigated following large sets of training trials, after at least 99 rotated-cursor trials [7, 10], little is known about how these aftereffects change across a shorter time frame. A recent study by our collaborators [14] looked at both reach aftereffects and proprioceptive recalibration across shorter blocks of time. They found participants’ reach aftereffects were significantly deviated after only 5 training trials and this deviation continued to increase until approximately the 40^th^ trial, after which it plateaued. Proprioceptive recalibration occurred much slower and was not significantly different from baseline performance until the 70^th^ trial. This slow change in sensory estimates is consistent with a study by [13] that measured proprioceptive estimates of hand motion intermittently throughout training with a curled force field. Their participants significantly recalibrated their sense of felt hand motion after 76 force-field training trials. Unlike [14], they did not measure aftereffects intermittently but only looked at null field effects after the completion of training. In these two studies, a “perceptual” task was used where participants reported (using a two-alternative force choice; 2-AFC) if their unseen, robot-led hand was felt to be located left or right of a reference marker (or the body midline). These perceptual measures are robust, yet they may not be able to capture the full extent of the change in felt hand position/motion since the tasks require 50+ trials. Both [13] and [14] show significant decay in motor learning following each block of perceptual tasks. To achieve denser sampling of these changes, we opted to use another method of measuring felt hand position, *proprioceptive-guided reaches* that has been extensively used in our lab [3, 15] and demonstrates similar changes following visuomotor adaptation as seen with the other perceptual measure [16]. The use of proprioceptive-guided reaches will allow us to compare the two processes at earlier time points and allow for high-resolution analysis of the rates of change.

Moreover, we will look at reach aftereffects and proprioception at consistent time intervals within training. While the recent studies discussed above provide great insight into the time courses of reach aftereffects and proprioception, in both studies the number of training trials was not evenly distributed between measures of proprioception (and reach aftereffects). Also, proprioception has yet to be tested in a way that can be directly related to reach aftereffects, making it difficult to be able to compare the time course and stability of sensory and motor changes. It is possible that sensory and motor changes follow different time courses, perhaps analogous to a proposed multi-rate model responsible for short term motor learning [17]. Using this multi-rate model and the paradigm designed to capture the two time courses (sensory and motor changes), it was found that the explicit component of learning follows the fast model while the implicit component resembles the slow process [18]. Therefore, we conducted a study to investigate the progression of these potentially distinct implicit measures; reach aftereffects and proprioception.

Motor and sensory changes have been shown to last at least one day following reach training to both a visuomotor rotation and velocity dependent force-field paradigm. Moreover, many studies have shown retention of reach adaptation after days, and even a year after visuomotor training [19], although retention of learning is usually measured with regards to faster re-learning [2, 20, 21, 22, 23]. Only a couple of labs have investigated the persistence of sensory changes. Specifically, our lab has previously shown that both reach aftereffects and proprioceptive changes do persist 24 hours after training, with a 45° cursor rotation [24]. Similar retention of a shift in perceived hand motion was found 24 hours after training with a curled force-field [12]. The degree to which these changes (particularly sensory changes) remain after a week is unknown, nor is it known if the remaining changes interfere with learning an opposite rotation. Individuals’ initial performance has been shown to be worse when learning an opposing visuomotor rotation 24 hours after learning the first rotation [2]. Interference after a week has been investigated with velocity-dependent force fields, but only in terms of learning rate, as no measurement of aftereffects or sensory changes were taken [2]. The current study will be the first to assess retention and potential interference of these sensory changes after a full week.

## Methods

### Participants

This experiment was completed by 41 right-handed healthy adults (mean age = 20.57 years, range = 17–42 years, males = 10). Participants were either lab members (naïve to the purpose of the study) or were recruited using the undergraduate research participant pool at York University; the latter group was given course credit for participation. All participants provided written, informed consent. Procedures were approved by the York Human Participants Review Sub-committee.

### Apparatus

**Fig 1:**
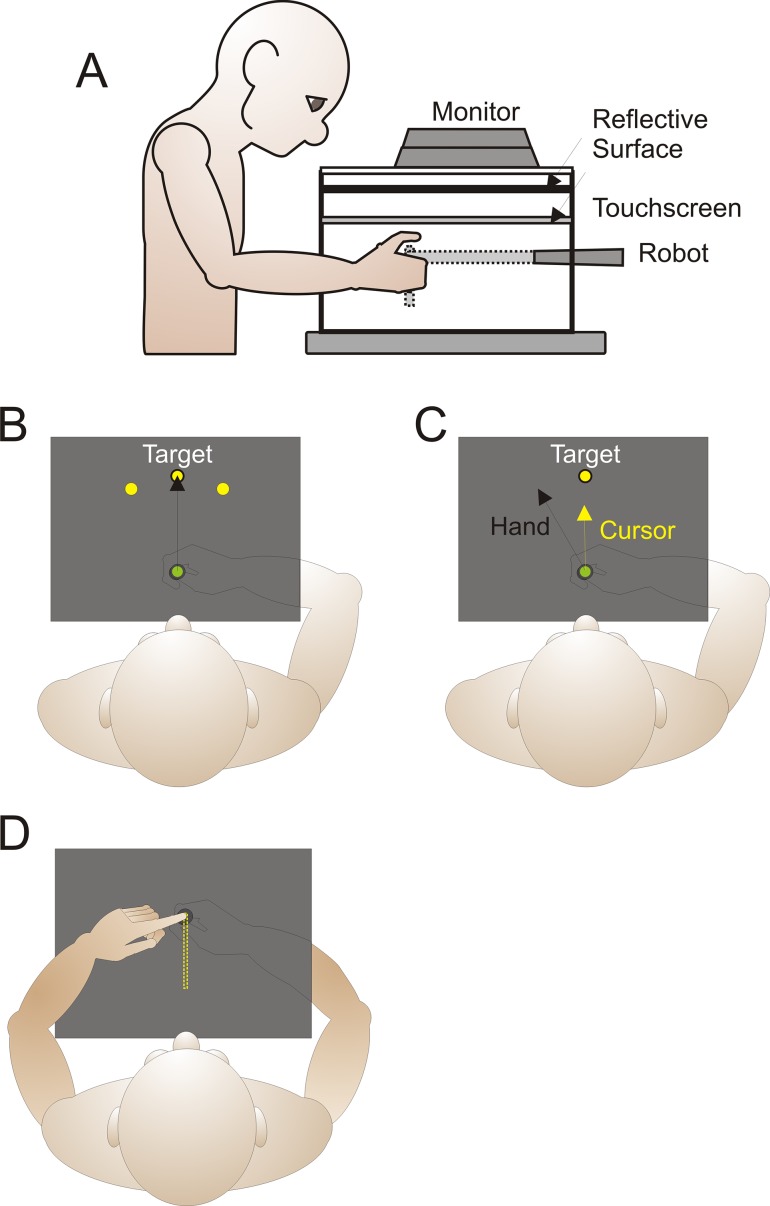
Experimental set up and design. *A*: Side view of the experimental set. *B-D*: Top views of task specific set ups. *B*: The home position was represented by a green circle with a 2 cm diameter; it was located 20 cm from the participants’ body midline. The home position was only visible prior to the appearance of the target at the beginning of the trial; it also reappeared after each trial. Each target was represented by a yellow circle with a 2 cm diameter located radially 12 cm away from the home position. The participants reached to three targets located at 60°, 90° and 120° in polar coordinates. *C*: During rotated reach training the cursor was deviated 30° CCW or CW (only CCW shown) with respect to the start location. *D*: In the proprioceptive guided reaching task, the robot passively moved (the yellow dashed pathway) the participants’ right adapted hand to one of the three target locations. The participants then used their left untrained hand to locate their right hands felt location.

A view of the experimental set-up is provided in Fig 1. Participants sat in a chair that could be adjusted with respect to height and distance from the display so that they could comfortably see and reach to each of the target locations presented on a reflective screen (Fig 1A). With their right hand, participants held a vertical handle on a two-joint robot manipulandum (Interactive Motion Technologies Inc., Cambridge, MA, USA), such that their thumb rested on top of the modified handle. The reflective screen was mounted on a horizontal plane 18 cm above the two-joint robotic arm. Visual stimuli were projected from a monitor (Samsung 510 N, refresh rate 72 Hz) located 29 cm above the robotic arm such that images displayed on the monitor appeared to lie in the same horizontal plane as the robotic arm. A 43 cm (length) × 33 cm (width) × 0.30 cm (height) touchscreen panel (Keytec Inc., Garland, TX, USA), with a resolution of 4,096 × 4,096 pixels was horizontally mounted 2.5 cm above the robotic arm to record reach endpoints (made with the left hand) made to proprioceptive hand-targets (described in detail later). The lights were dimmed; the subject’s view of their training (right) arm was blocked by the reflective surface and a black cloth that was draped over their right shoulder. The view of the left, untrained hand was not concealed, and lit by a small lamp, so that the left arm was visible during the proprioceptive localization task, and any errors in reaching to the unseen target hand could not be attributed to errors in localizing the left reaching hand, thus removing the potential of interlimb transfer influencing the results.

#### Reach Stimuli

In both the reach training and no-cursor reach tasks there were three possible targets, each represented by a 1 cm diameter yellow circle. The targets were located radially, 12 cm from the home position at 60°, 90° and 120° in polar coordinates (Fig 1B). Fig 1B and 1D displays the different tasks and target locations used throughout the experiment. The cursor, used to represent the participant’s hand, was a green circle 1 cm in diameter. The home position was only visible briefly before the target onset and was located 20 cm in front of the participant at their body midline. The home position and the target were never shown at the same time. The robot kept the participants’ adapted, right hand locked at the home position during the intertrial interval of 500 ms.

#### Proprioceptive Stimuli

For proprioceptive hand localizations, the right hand served as a target and was moved by the robot to one of the three target locations previously described. A beep then signaled participants to use their untrained, visible, left hand to point on a touchscreen to where they felt their trained right hand to be (Fig 1D). Once the touchscreen registered their finger touch, the right target hand was allowed to move back to the home position along a robot-constrained path [9], while only the home position was visible. The hand was then locked at the home position for 500 ms before it was passively moved to the next target site. We use this method to measure proprioception instead of a 2-AFC procedure as it takes far less time and has been shown to be equally effective [16]. The left hand was always visible to avoid interlimb transfer and to ensure that any systemic changes in reaching to the right target hand were not due to problems localizing the left reaching hand.

### Procedure

The experiment occurred on two separate testing days with two testing conditions the first day and one condition completed the second day. The testing days were exactly one week apart. Participants trained to reach with a cursor (Fig 1C) and performed two additional interleaved tasks meant to measure changes in reaches and changes in felt hand position. Specifically, they reached to the same targets without a cursor and located their passively placed right hand by reaching with the left hand (Fig 1D).

**Fig 2:**
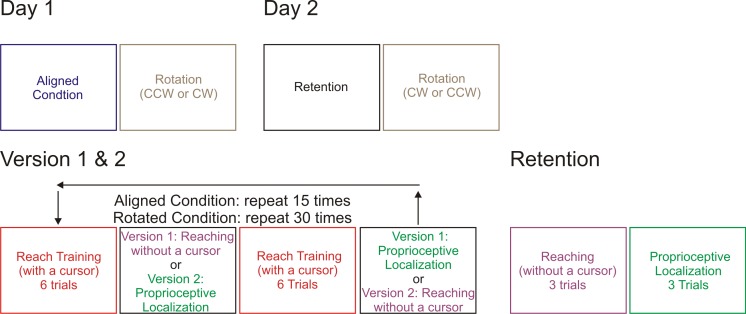
Breakdown of experimental set up. *Day 1 and Day 2* illustrate what conditions participants experienced on which testing day. All participants started with aligned cursor training on Day one then learned one of the two rotations (CCW or CW). All participants then returned one week later for *Day two* and completed the *retention* condition (which is described in further detail to the right) and learned the other rotation direction(CW or CCW). All participants completed the same tasks throughout training, but there were two tasks orders, counterbalanced across participants *(version 1 and version 2)*. Participants completed a total of 270 trials during the aligned-cursor condition and 540 trials during the rotated cursor condition.

Reach training was conducted with an aligned cursor and a rotated-cursor condition, the cursor was rotated by 30°, either clockwise (CW) or counter clockwise (CCW). Participants were randomly assigned to one of the four testing groups: CW rotation first and version one of task order, CW rotation first and version two, CCW rotation first and version one, CCW first and version two. Eleven participants were in the first testing group and ten were in each subsequent group. In order to test retention, we had participants come back one week later for a retest on the no-cursor reaches and proprioceptive localization. We then had them repeat the rotation condition from Day 1, with the opposite rotation (Fig 2. Day 2: brown box). This additional training with the opposite rotation allowed us first to test for anterograde interference, which we did not find. Thus, in the absence of interference, we combined the data from both sessions for a better analysis of the time course of the effects.

The retention condition only consisted of two tasks (six total trials), no-cursor and proprioceptive localizations (in that order) to the same three targets as previously described, regardless of version (Fig 2 bottom-right). These retention trials were completed upon the participants’ return on Day two, before they began training with the opposite cursor rotation.

#### Reach-training task (red boxes in Fig 2)

During the reach training task (Fig 1C and Fig 2 red boxes) participants reached to the targets while holding onto a robot manipulandum with their right hand (Fig 1A). Before the first target was presented the word “cursor” was displayed on the reflective surface for 1000 ms. They then reached to the presented target with either an aligned (Fig 2 Day 1) or rotated cursor (Fig 2 Day 1 and 2) representing their hand location. The participants were required to obtain the target in each trial and were instructed to move their hand-cursor as quickly and accurately as possible to the visible target. These trials began with the hand locked in place at the home position marker, which disappeared once the target appeared. At the time, the participant’s hand was released and they were able to reach to the target. A trial was considered complete when the cursor overlapped the target for 300 ms. At that point both the target and cursor would disappear and the home position marker would reappear; the participant actively moved their hand back to this location along a robot-constrained pathway, with only the green home circle being visible. Upon returning to the home position, the right hand was locked in place for 500 ms before the next trial began. The targets were presented in a pseudo-random order so that no target was repeated before all three were shown, and were grouped into blocks of 6 trials. During the aligned-cursor training (blue box) there were 15 blocks (180 trials in total), and during the rotated-cursor training conditions (black boxes) there were 30 blocks (360 trials in total).

#### No-cursor Reaching task (purple boxes in Fig 2)

Reaching without a cursor before and after training with a rotated cursor is the traditional assessment of reach aftereffects (Fig 2 purple boxes). After completing six or twelve reach training trials participants completed three no-cursor reaches to the same three targets. The words “no-cursor” were displayed prior to each block of three trials for 1000 ms. The participants were required to reach to the target in the same manner as the prior task, stop when they believed their unseen right hand was directly under the yellow target, and hold their hand still for 300 ms to complete the trial. At that time, the target disappeared and the home position appeared, cuing participants to return their hand to the home position just as in the reach training task. The participants completed three no-cursor trials in a row, one to each of the three targets. During aligned-cursor training (Fig 2 blue box), there were 15 blocks of 3-trials (45 no-cursor reach trials in total), while during the rotated-cursor training conditions (Fig 2 black box), there were 30 blocks (90 trials in total), and only 3 total trials during the retention condition (Fig 2 brown box). This small number of trials allowed us to capture the initial retention, before the usual decay associated with washout.

#### Proprioceptive localization task (green boxes in Fig 2)

The proprioceptive localization task differs from the previous two tasks in that there was no visual stimulus, i.e. no visible target (Fig 1D and Fig 2 green boxes). Participants were directed with the words “reach to hand” prior to each block of three trials. During proprioceptive localizations the trained right hand was used as the target, which was passively moved by the robot to one of the three visual target locations (duration of 650 ms for the 12 cm displacement; see 9). A beep signaled participants to use their visible left index finger to point, on a horizontal touch screen, to the felt location of the right target-hand, more specifically the location of the thumb that rested on top of the manipulandum. Once the left-hand reach endpoint was registered on the touch screen panel, the robot released the right hand and the home position marker reappeared. The participant then returned their left reaching hand to the side and actively moved their right target hand back to the home position along a constrained pathway, just as in the previous two tasks. During aligned-cursor training (Fig 2 blue box), there were 15 blocks of 3-trials (45 proprioceptive localization trials in total), while during the rotated-cursor training condition (Fig 2 black box), there were 30 blocks (90 trials in total), and only 3 total trials during the retention condition (Fig 2 brown box).

There was no time limit imposed on any of the tasks. Participants were instructed to reach quickly and had generally uniform performance times (no participant took more than one and a half hours and none took less than an hour and fifteen minutes). Reach adaptation does not differ when participants are given lots of time to make corrections in order to acquire the target versus when they are told to quickly slice through the target (no correction) [25]. This means that allowing a participant to take as long as they need, or requiring them to complete the task in a certain time will result in the same amount of adaptation.

The first day (Fig 2 “Day 1”) of the experiment took approximately 70 minutes of testing and the final day (Fig 2 “Day 2”) was roughly 45 minutes long.

### Data Analysis

Our aim was to investigate both motor and sensory changes across time, specifically the changes in no-cursor reaches and change in hand localization, measured in consistent intervals during reach-training. For both of these, we looked at endpoint error. Proprioceptive localizations were based on the angular endpoint error as provided by the difference between the movement endpoint and the responses on the touchscreen, while no-cursor reaches (to assess reach aftereffects) were angular endpoint deviations of the hand from a straight line from home-position to the visual target. For completeness, we also assessed the training performance (reaches with cursor) by computing angular deviation of the hand (robot) movement at peak velocity relative to target direction. There were no endpoint errors for these trials since participants had to attain the target to the complete the trial.

As we wanted to test these changes across time, our main factor of interest was Block (Aligned, First rotated, Final rotated), with Rotation (CW or CCW) as a secondary (less important) factor. In order to make the data from each rotation (CW and CCW) comparable in our analyses, we had to normalize our data. Since all our measures were angular deviations, we multiplied the CW data for all three measures by -1 so that changes were in the same direction for both CCW and CW rotations. This also allowed us to collapse our data and analyze the changes across testing days. While our main analyses used the factors Block and Rotation, we also ran a separate set of ANOVAs that also included the factors Version (trial type order within the task) and Order (CW trained first or CCW trained first). Since those factors did not interact with Block and hence had no influence on the results, these ANOVAs are not reported. The ANOVAs that we do report (mixed ANOVAs using Block and Rotation) were identical for all three measures; and each showed that Rotation had no effect (did not interact with Block), for brevity these were also omitted.

Since there was an effect of Block in all these analyses, we then wanted to tease apart the changes across time, and we then ran three more mixed ANOVAs on each of the three measures. Each of these only included two Blocks, as well as the factor Rotation. We compared the Aligned block, with the First rotated block, to see if changes occur quickly, and we compare the First rotated with the Final rotated block to see if further change occurs between the start to the end of training. In order to counter multiple testing we use an alpha level of p = .01 for these sets of three ANOVAs. All values are reported with a Greenhouse Geisser correction. With these analyses we can get a complete picture of several changes occurring across time in a visuomotor rotation adaptation paradigm.

To answer our secondary questions about retention and interference; we completed further ANOVA’s to detect their existence. The retention block of trials (no-cursor and proprioceptive localizations) were compared to the Aligned-block and the Final-block of rotated trials from the first day of training, within each task. A significant difference between the aligned block and retention block would suggest that changes in hand localization and reach aftereffects had been retained from Day 1, while a significant difference between the retention block and final training block on Day 1 would suggest that retention was less than complete. To test for interference, we first tested whether the change in reaches and proprioceptive localization in the first block of Day 1 and Day 2 differed, specifically if errors on Day 2 were significantly larger than those on Day 1. But to determine whether interference resulted in a slower learning rate for Day 2 we compare the first five blocks of trials from Day 1 to the first five blocks of trials from Day 2, for each task type to test for Day*Block interactions.

## Results

### Reach Training

**Fig 3:**
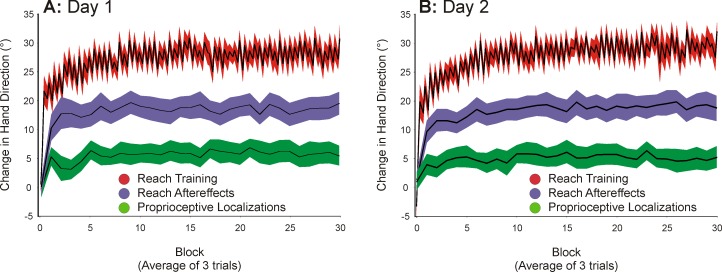
Mean change (relative to baseline, marked as zero) in angle at max velocity for reach training (red) and angular endpoint error for both no-cursor reaches (purple) and proprioceptive localizations (green), plotted for each separate testing day. All are plotted across blocks of 3 trials (averaged across trials and participants). These reach and proprioceptive deviations were flipped, so as to shift in the same direction, for ease of viewing for Day 1 *(A)* and Day 2 *(B)* of testing. Participants do show slight forgetting after each block of no-cursor and proprioceptive localizations, this can be seen in the jagged pattern of learning in the reach training. The black lines within the colored curves represent the block means while the colored areas represent a 95% confidence interval.

**Fig 4:**
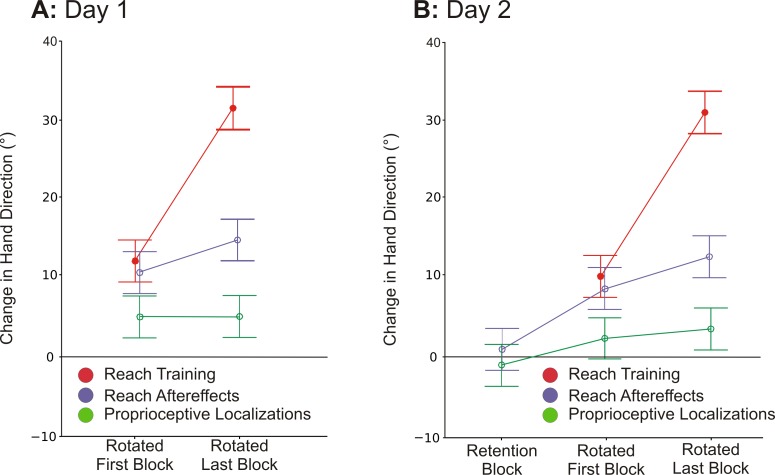
Mean changes in hand location at peak velocity and at reach endpoint for each respective task as in Fig 3, for the first and final block of training in Day 1 *(A)* and in Day 2 *(B)*, relative to aligned-training baseline. Error bars represent +/- 1 standard deviation from the average. The line at zero represents baseline performance.

We first confirmed that participants had altered their hand movements to obtain the target during the imposed rotations. Fig 3 shows these changes in hand movement direction (in red); we plotted hand direction (rather than cursor direction) in order to compare them with the no-cursor reaches that were systematically interspersed with training trials. During the rotated conditions participants initially made large hand-cursor errors (not shown) since their hand direction (Fig 3, red line) was still aiming toward the target. But with training the cursor-reach errors became smaller, as can be seen by increases in hand direction deviation (Figs 3 and 4). This increase in hand direction deviation across training was significant (Day 1: F(1.90, 74.19) = 238.63, P < .001; Day 2: F(1.83,71.54) = 184.62, P < .001). More specifically hand movement directions were significantly deviated at the start of training with a rotated cursor, in that the initial block of three rotated trials (Fig 4) were significantly different from those of the final block of aligned trials (Day 1: F(1,39) = 81.13, P < .001; Day 2: F(1,39) = 33.40, P < .001). These hand movement direction deviations continued to grow throughout training as the first block of three rotated trials were significantly different from the final block of rotated trials, shifting from 10° to 29.4° on Day 1, and from 8° to 28.8° on Day 2 (Day 1: F(1,39) = 144.47, P < .001; Day 2: F(1,39) = 140.69, P < .001). As a final indication that participants adapted successfully in this experiment, we also found no difference in the cursor direction (not shown) at the end of rotated-cursor training (final trials) with those produced during the aligned reach training (Aligned vs. Final block: Day 1: F(1,39) = .01, P = .905; Day 2: F(1,39) = .03, P = .872).

#### Interference

To identify if there were any differences in learning on the second testing day which were attributable to interference, we compared the first block of cursor-rotated training trials for each separate testing day (Fig 3A vs. 3B, initial part of each curve). We found no significant difference (F(1,39) = 1.05, P = .312). We also compared the changes across the first five blocks as a function of Day, and found no significant interaction (F(3.48,139.02) = .50, P = .708). This suggests that initial learning rate did not differ as would be expected with interference. Not surprisingly, the amount of adaptation by the final block of trials for both days was equivalent, with no significant difference across the two days (F(1,39) = 2.19, P = .147).

### Reach Aftereffects

Mean direction endpoints for no-cursor hand movements produced during reach training are shown in purple in Fig 3. To determine whether there was a significant difference during the early phases of training (first block, after only 6 or 12 reach training trials), as well as during the late phases of training (the final block), we compared these endpoints errors with no-cursor reaches made at the end of aligned training (Day 1: F(1.98,77.32) = 77.35, P < .001; Day 2: F(1.85, 72.18) = 53.19, P < .001). When looking at Day 1 of rotation training (Fig 4A), we found that no-cursor reach endpoints shifted by 10.5° after only one block of training (align vs. initial: F(1, 39) = 81.30, P < .001), and by 14.5° at the final block (align vs. final: F(1,39) = 132.31, P < .001). This same pattern of results was found for Day 2 (Fig 4A), with participants’ reach endpoints being deviated by 8.3° during the initial block of rotated training (align vs. initial: F(1,39) = 63.10, P < .001) and by 12.4° by the final block of trials (align vs. final: F(1,39) = 83.44, P < .001). We also see significant subsequent learning throughout rotated training (initial rotated block vs. final rotated block: Day 1:F(1,39) = 11.05, P < .002; Day 2: F(1,39) = 10.47, P < .003), which indicates that even though there was substantial, immediate learning, there was still some motor adaptation occurring throughout the rest of training. As with other studies [14, 26, 27], these final no-cursor reaches were less deviated than cursor-reaches during training overall (red line in Fig 3). We report results for endpoint errors, rather than angle at peak velocity, so these errors could better match the endpoint errors measured for proprioceptive localizations. However, we found similar results when we analyzed angular errors at peak velocity. In short, we find that no-cursor reaches change very quickly, achieving almost one-third of the distortion after a single set of training trials.

#### Retention

One week after the first testing session participants returned and immediately completed three no-cursor reaches. These are displayed as angular endpoints in Fig 4B (purple). These reaches (normalized so deviations appear in the same direction regardless of original cursor deviation direction) show no evidence of retention. The deviations of the no-cursor reach endpoints (.96°) were very similar to those produced after training with an aligned cursor (.22°) on Day 1 (F(1,39) = 2.64, P = .113) and not close to the shift produced immediately following training, i.e. 14.5° (F(1,39) = 123.04, P < .001). That is, we found no retention of changes in no-cursor reaches one week after training.

#### Interference

We found no evidence of anterograde interference when learning an opposing rotation a week later. Specifically, initial reach aftereffects on the First Block on Day 2 (Fig 3A vs. 3B, initial part of each curve) did not differ from those on Day 1 (F(1,39) = 2.26, P = .141). We also found no evidence suggesting that the early phases of learning were affected, in that the first five blocks of reach aftereffects did not differ across the two days (no Block*Day interaction, F(3.43,137.17) = .34, P = .821).

### Proprioceptive Localization

Shifts in localizing the position of the unseen, right target hand using proprioception only are depicted in green in Figs 3 and 4 (averaged across blocks like the no-cursor reach endpoints). Shifts in localization fall naturally in the opposite side of no-cursor reaches since a “CW” error in this case indicates that the hand felt shifted CCW, however results are plotted in the same direction for ease of viewing. Participants were fairly accurate at localizing their unseen, right hand during aligned cursor training, such that they were on average less than a degree away from the hand-target (.32°), regardless of the cursor rotation that was subsequently introduced (CW or CCW). We found a significant effect of Block, suggesting that training with a rotated cursor led to proprioceptive recalibration (Day 1: F(1.97,76.83) = 14.16, P < .001; Day 2: F(1.70,66.20) = 4.08, P = .027). Upon further analysis it was evident that even the first set of rotated-cursor training led to a significant shift in proprioceptive localization (Fig 4) by an average amount of 5.20° following reaches on Day 1 and 2.50° on Day 2, although this initial shift on Day 2 was not significantly different than during the aligned training (aligned vs. initial: Day 1: F(1, 39) = 22.43, P < .001; Day 2: F(1,39) = 2.76, P = .104). Nonetheless, by the end of training, their localizing errors were significantly shifted from the aligned trials for both Days (align vs. final: Day 1: F(1,39) = 19.88, P < .001; Day 2: F(1,39) = 6.53, P = .05). However, these changes observed at the end of the training session with the rotated cursor were not significantly different from changes observed just after the rotation was introduced, with a final shift of 5.5° on Day 1 and 3.7° on Day 2 (initial vs. final: Day 1: F(1,39) = .04, P = .84; Day 2: F(1,39) = 1.67, P = .20). To sum up, proprioceptive recalibration occurred rather quickly and did not significantly increase over training.

#### Retention

We tested for retention of any proprioceptive recalibration by comparing whether participants’ estimates of felt hand position for a single block of trials produced one week after rotated training (Fig 4B) differed from those produced during aligned training. We did not find any evidence of retention (F(1,39) = 1.61, P = .211).

#### Interference

To determine if there was any interference of the originally learned rotation in changes of felt hand position following training on the second testing day, we compared the first block of rotated training from Day 1 to that of Day 2 (Fig 3A vs. 3B, initial part of each curve). We found no significant difference between days (F(1,39) = 3.67, P = 0.062). In addition, we compared the first five blocks from Day 1 to the same blocks from Day 2, and found no significant interaction (F(3,119.83) = 1.71, P = .169), once again confirming that there was no difference in initial learning rate.

## Discussion

Our study shows that reach aftereffects and proprioceptive recalibration appear very rapidly during training with a visuomotor rotation. After only six or twelve rotated-cursor training trials, participants’ no-cursor reaches shifted by 9.4° (averaged across both days). These reach aftereffects increased by an additional 4.1° over the course of training to 13.5°. Surprisingly, participants’ sense of felt hand position was also found to have shifted 3.9° in the direction consistent with adaptation after the initial training block, increasing only slightly to 4.6° by the end of 180 rotated training trials. Changes in proprioception were smaller than changes in no-cursor reaches, suggesting that these motor and sensory adjustments may involve independent processes. These changes were not retained a week later and led to no interference when learning a rotation of the opposite direction.

### Time Course

Many studies have looked at changes in perturbed reaches produced throughout training with a visuomotor rotation, such as [4]. In nearly all cases, cursor-reaching errors were initially large but gradually decreased, saturating to near baseline levels within 20–30 trials per target [4, 6, 19]. The results of rotated cursor reaches in the current study are consistent with these previous reports given that our participants also adapted to the cursor rotation within 30 trials for three nearby targets, despite reach training being interrupted by the other tasks.

After confirming our participants had adapted, our main objective was to investigate the progression of motor and proprioceptive recalibration (i.e. their learning curves). While many studies have measured reach aftereffects, as a hallmark of motor learning, they usually do so after a large training set (at least 100 training trials). Likewise, our previous measures of proprioceptive recalibration were always preceded by at least that many trials. Thus, it has remained unclear when and how quickly during training reach aftereffects and proprioceptive recalibration emerge. This kind of information would provide insight into the types of mechanisms and processes associated with visuomotor learning. We found that these changes emerged surprisingly quickly during training (after 6–12 trials of cursor-training), with proprioceptive recalibration reaching a plateau and reach aftereffects increasing with further training. These differing adaptation time courses lead us to believe the mechanisms driving these systems may be separate.

Some researchers have attributed the time course or pattern of learning *while reaching with the perturbation* as reflecting two separate systems, a fast and a slow system [17]. The fast system responds strongly to errors, contributing to quick changes in hand movements during the initial rotated-cursor training trials. Contrary to the fast system, the slow system is less sensitive to error, proceeds more gradually, and persists for a longer period of time. The fast learning phase was initially suggested [28, 29] and more recently tested and modeled by [18] to reflect the explicit or conscious effort to reduce aiming errors, while the slow system reflects a more implicit contribution to adaptation. The fit (fast/slow distinction) by [18] was weaker when the trained targets spanned only one quadrant rather than the full 360° space. In that case the implicit component of learning, normally associated with aftereffects, did increase quite quickly and slowly increased further with training. The time course of their indirect measure of implicit learning resembles the results of our implicit learning measures, i.e., reach aftereffects and proprioceptive recalibration, measured throughout training for targets that also only spanned 60 degrees. We did not design this experiment with an opposing training task immediately following the first rotation and currently have no way of conducting a clamp trial for our localizing measure (as used by 17, 18). Therefore, we cannot say for sure whether both aftereffects and proprioceptive recalibration follow a slow-learning phase model.

### Proprioceptive and motor recalibration

The time courses described above are those for trials with the perturbation in place. Our attempt was to look at those produced for reach aftereffects and proprioception; as few have investigated these changes during adaptation and instead measure recalibration after training. Previously no-cursor reaches had only been measured once after every two sets of reaches to 8 radial targets while training with a 30° CW rotation for 300 trials [30]. They found reach aftereffects shifted by 15° very early on, specifically by trial 17 and after reaching twice to each target for their implicit group which resembles our training groups. These reach aftereffects did not appear to increase substantially after 300 trials, but were deviated by another 5° by the end of training. Our lab has also found that reach training, either during the short time scale of the current study, or during even longer time scales [5, 7, 10], does not seem to lead to larger reach aftereffects.

Training with a visually distorted cursor, rotated or translated, not only leads to deviated reach movements (i.e. reach aftereffects) but also perceived hand position becomes more deviated [7, 8, 9, 10, 11]. Specifically, we have shown that sensory changes in hand position become deviated by 15–20% of the imposed distortion following approximately 100 trials of reach training. In the present study, the proprioceptive recalibration observed was also about 15% of the rotation, but this change was obtained very early in training, and did not increase much further. We found a similar lack of increase in proprioceptive recalibration (and reach aftereffects) when measured after a series of larger training sets of 99 trials (30° cursor rotation repeated three times; 10). Likewise, using the same rotation size, but providing only terminal or endpoint cursor feedback during training led to progressively more proprioceptive recalibration [7]. Specifically, [7] found that felt hand position changed by about 3.4° and was not significant after the first set of 99 training trials, but did increase significantly to 7.4° by a third block of training. Thus, the rapid recalibration of hand proprioception in our study seems to require continuous cursor feedback, which allows for more sensory information to be utilized. This is consistent with our other studies showing that it is the discrepancy between the senses, rather than errors in movements, that drive this sensory change [8, 31].

A recent article by collaborators [14] found that proprioception slowly recalibrated, with proprioceptive shifts not being significantly different from baseline until after 70 rotated training trials (30° CW visuomotor rotation). They implemented a protocol similar to that of [13], which tested for somatosensory changes during force-field adaptation. They also found that this sensory shift required at least 70 trials training with the force field. Both studies used a 2-AFC perceptual task to obtain estimates of proprioceptive recalibration and decay in learning can be seen after each round of 50 perceptual trials. Many of our studies on this topic use a similar perceptual (2-AFC) method as mentioned above, and we appreciate the advantages it offers. However, we opted to use proprioceptive guided reaches to the adapted hand instead in the current study, as each trial would provide a proprioceptive estimate of the change in felt hand position. Given the advantage of this method with respect to time required to complete the task, we can assess changes in hand proprioception in only three trials, many times (e.g. 30 times) and equally dispersed throughout training. It is possible that our method may be more sensitive to detecting the onset of proprioceptive recalibration, as it would occur in most adaptation paradigms. This method also allows for these changes in hand proprioception to be compared to changes in reach aftereffects almost in real time, creating comparable time courses for both motor and sensory changes.

The previously mentioned study [14] also measured reach aftereffects just prior (and after) each block of 2-AFC perceptual tasks. Their block of six no-cursor reaches did show a similar rate of change to what we found, with a significant shift after the first block of five training trials and near-saturation after an additional five trials. The similarities between reach aftereffects lead us to believe the quicker method of measuring proprioception is more effective. This is as it allows for collection of more trials without interfering with learning and does not seem to interfere with the progression of aftereffects.

### Retention and interference

Retention of motor recalibration has generally been measured by looking for savings or re-learning rates [23, 32, 33, 34]. However, since we wanted to compare retention for both motor and sensory recalibration, we measured retention by testing for sustained changes in reach aftereffects and felt hand position. We used a similar method to investigate retention for both motor and sensory changes 24 hours after reach training with a 45° CCW-cursor [24]. In that study, after 150 continuous training trials, no-cursor endpoint errors were deviated by 11.8° or 26% of the distortion introduced during training and proprioceptive estimates (using a perceptual task) were also deviated by 4.5° or 10%. During testing 24 hours later, 46% of reach aftereffects and 72% of the proprioceptive recalibration achieved were retained. The current study found slightly larger reach aftereffects (43% of imposed rotation) and proprioceptive deviations (15% of the rotation) during and following training. However, we found no retention of either task the following week. This is contrary to [19] who found participants’ aftereffects were still significantly deviated a year after training (59%–91% of the 40° visuomotor rotation). They used a joystick for their visuomotor adaptation study so equipment-specific memory may have contributed to the persistence of the retention. Another lab has tested the effect of force-field adaptation on the perception of unseen hand motion [12] and found that sensory changes were retained 24 hours later. Thus, results of our two studies (along with 12), suggest these small but robust sensory changes following motor learning are retained for at least 24 hours, but are lost within a week.

Given that we found no retention for both measures, it is not surprising that we found no anterograde interference after one week. Interference is usually measured by re-testing a visuomotor rotation following adaptation to an opposing perturbation (Task ABA paradigm). Results typically show that initial errors and learning rate are worse than those when first exposed to the original perturbation (retrograde interference, Task B caused forgetting of task A). Thus, there are no savings as would normally be found when there is no interference, i.e. when there is no task B present between test and retest [2, 23, 34, 35]. In our study, we did not retest the original cursor rotation, but instead attempted to measure anterograde interference; that is participants’ performance on the opposite rotation a week later (after a short retention task that did not provide any visual feedback). Initial performance (training errors or learning rate) during reach training trials did not differ across testing days, which loosely suggests no anterograde interference.

### Conclusion

After only 6–12 rotated-cursor training trials participants adapted their reaches and shifted their felt hand position. Reach aftereffects increased to an even larger degree of deviation with further training, whereas proprioceptive recalibration remained stable after the first block. When participants returned one week later to learn the opposite rotation (CW vs. CCW) there was no evidence of retention or interference for either reach aftereffects or proprioceptive recalibration. Further investigation is required to identify how these two measures of implicit learning work together to influence overall motor output and adaptation.
